# Unbearable suffering and requests for euthanasia prospectively studied in end-of-life cancer patients in primary care

**DOI:** 10.1186/1472-684X-13-62

**Published:** 2014-12-23

**Authors:** Cees DM Ruijs, Gerrit van der Wal, Ad JFM Kerkhof, Bregje D Onwuteaka-Philipsen

**Affiliations:** Department of Public and Occupational Health, VU University Medical Center, EMGO+ Institute, Expertise Center for Palliative Care, van der Boechorststraat 7, 1081 BT Amsterdam, The Netherlands; Department of Clinical Psychology, VU University, EMGO+ Institute, van der Boechorststraat 1, 1081 BT Amsterdam, The Netherlands; Primary Care Center De Greev, Grevelingenstraat 10, 3522 PR Utrecht, The Netherlands

## Abstract

**Background:**

An international discussion about whether or not to legally permit euthanasia and (or) physician assisted suicide (EAS) is ongoing. Unbearable suffering in patients may result in a request for EAS. In the Netherlands EAS is legally permitted, and unbearable suffering is one of the central compulsory criteria. The majority of EAS is performed in cancer patients in the primary care practice. In around one in every seven end-of-life cancer patients dying in the primary care setting EAS is performed. The prevalence of unbearable symptoms and overall unbearable suffering in relationship to explicit requests for EAS was studied in a cohort of end-of-life cancer patients in primary care.

**Methods:**

A prospective study in primary care cancer patients estimated to die within six months was performed. Every two months suffering was assessed with the State-of-Suffering V (SOS-V). The SOS-V is a comprehensive instrument for quantitative and qualitative assessment of unbearable suffering related to 69 physical, psychological and social symptoms in five domains.

**Results:**

Out of 148 patients who were asked to participate 76 (51%) entered the study. The studied population were 64 patients who were followed up until death; 27% explicitly requested EAS, which was performed in 8% of the patients. The final interview per patient was analyzed; in four patients the SOS-V was missing. Unbearable symptoms were present in 94% of patients with an explicit request for EAS and in 87% of patients without an explicit request. No differences were found in the prevalence of unbearable suffering for physical, psychological, social and existential symptoms, nor for overall unbearable suffering, between patients who did or who did not explicitly request EAS.

**Conclusions:**

In a population of end-of-life cancer patients cared for in primary care no differences in unbearable suffering were found between patients with and without explicit requests for EAS. The study raises the question whether unbearable suffering is the dominant motive to request for EAS. Most patients suffered from unbearable symptoms, indicating that the compulsory criterion of unbearable suffering may be met a priori in most end-of-life cancer patients dying at home, whether they request EAS or not.

## Background

Unbearable suffering is considered an important motive for patients requesting euthanasia and/or assisted suicide (EAS) [1]. Seven countries and states have legalized EAS, or permit EAS under existing law [2]. The presence of unbearable suffering, as assessed by a physician, is a central criterion for EAS in the Netherlands, Belgium and Luxembourg [3–6]. Further compulsory criteria to legally allow EAS include a voluntary and well considered request for EAS, prospect-less suffering, absence of reasonable treatment options and consultation with an independent physician [6, 7]. Terminal illness is not a compulsory criterion [6]. The legal model which applies in Switzerland, Oregon, Washington and Montana requires a voluntary and well considered request; unbearable suffering is not a compulsory criterion and only assisted suicide is allowed [2, 8, 9]. Terminal illness is a compulsory criterion in the U.S. states [2].

The Netherlands has a population of nearly seventeen million and 136.000 annual deaths. Annually some 40.000 patients die from cancer (28% of all deaths), 45% of whom die at home [10]. Primary care nationwide is provided by nearly 9.000 general practitioners (GPs), 57% of whom work part time [11]. A full time GP on average provides end-of-life care for a cancer patient nearly three times a year [12].

Performed euthanasia in the Netherlands has been studied in 5-year intervals since 1990 [13, 14]. In 2002 EAS was legalized. By 2005 the total number of explicit requests for EAS was 8400; 29% of all explicit requests for EAS were granted; 1.8% of all deaths were the result of EAS (n = 2410) [14, 15]. In 2010 the total number of explicit requests for EAS was 9100; 45% of all explicit requests for EAS were granted; 2.8% of all deaths were the result of EAS (n = 4050) [13, 16]. In the 2010 study 79% of all EAS patients has a cancer diagnosis. Eighty-eight percent of EAS cases were performed in primary care [13].

In end-of-life cancer patients dying in the primary care setting EAS is performed in around one in every seven patients [17]. EAS most frequently is performed in the estimated last two weeks of life [13]. Legal responsibility for EAS allotted to the medical profession changes the dynamics of interaction between patients and physicians [18]. Doctors face difficulties in assessing and responding to suffering and requests for EAS [1, 3, 19–23]. Patient directed research investigating unbearable suffering in relationship to whether an explicit request for EAS is made is scarce. In this study the presence and nature of unbearable suffering and its relationship to requests for euthanasia was investigated.

## Methods

### Design and population

The study was conducted in Utrecht, a city with about 235 000 people and 105 GPs. Those eligible for the study were terminal cancer patients expected to die within half a year and who were expected to live at home (most of the time) until death. They were cared for by a GP as the primary responsible physician. The GPs estimated survival, and clinical deterioration guided estimation of survival.

Forty-four GPs, representing 42% of the GPs in the city, 59% of whom worked part time, requested participation from eligible patients. A study coordinator organized the recruitment process, which included identifying all eligible patients in the care of GPs during the follow-up period. Baseline-characteristics of all eligible patients were registered. Within a week the baseline interview was administered to consenting patients. Follow-up interviews were administered every two months, or sooner based upon information by GPs that the condition of a patient had rapidly deteriorated. All interviews were at the patients’ residence. GPs were personally contacted every two months for follow-up data. When a patient died the treating physician was asked whether an explicit request for EAS had been made; a record of the date of the request was not part of the study design. Whether EAS actually was performed was not part of the initial physician-directed follow-up, because of the potential negative influence on recruitment related to enquiring about this sensitive subject. Transition to a hospice was not an exclusion criterion; in many Dutch hospices the GP remains responsible for palliative care. The interviewers were a physiotherapist (the study coordinator) and a GP (CR), both trained in interview techniques. The study protocol was approved by the Medical Ethics Committee at the VU University. Written informed consent was obtained from participants. The recruitment process is described in detail elsewhere [24].

Patient recruitment occurred from May 2003 until May 2006, follow-up continued until May 2007. There were 258 eligible patients. One hundred and ten patients were not requested to participate, in majority because their physical condition deteriorated so rapidly that their GP considered an interview too burdensome. Seventy-six out of 148 invited patients (51%) entered the interview study. Seventy-two patients refused to participate, in majority because of rapidly deteriorating physical condition and considering the interview too burdensome. In the 76 patients who entered the interview study the attrition rate was 8% (n = 6), caused by patients who stopped participating after one or more interviews. At the end of follow-up period 8% (n = 6) of the patients were alive, leaving 64 patients with follow up until death. In 60 patients at least one SOS-V interview was present; in four patients the interview was missing; the interviewer had considered the interview too burdensome and abandoned. In 33 patients the SOS-V was administered at least two times. Age, gender and type of cancer did not differ between the patients in and out of the interview sample. In January 2014 the GPs who had been addressed with a request for EAS were personally contacted to assess whether EAS had been performed. All GPs agreed to share this important information. Medical files were checked and dying trajectories were evaluated.

### Issues concerning the investigation of unbearable suffering

Unbearable suffering was defined as a subjective experience of suffering that is so serious and uncontrollable that it overwhelms one’s bearing capacity [25]. Unbearable suffering is a relative experience, which may be more or less present. Quantitative investigation of unbearable suffering provides the opportunity to compare patient populations. The measure of unbearable suffering needs to be differentiated from the intensity of symptoms; symptoms may be intensely present yet bearable, and vice versa [17].

Evaluation of unbearable suffering in relationship to specific disease indicates a systematic investigation of whether disease specific symptoms are present [26–29], followed by an assessment of whether symptom related unbearable suffering occurs. Suffering caused by co-morbidities, particularly if these result in unbearable suffering, may confound study outcomes and needs to be assessed. Investigation of the various physiological systems whether symptoms caused by co-morbidity occur and whether these symptoms result in unbearable suffering is indicated. Suffering reaches further than biomedical symptoms, it includes the consequences and meaning of disease for the various domains of life. These domains include practical daily functioning (functional domain), the perspective of self (personal domain), functioning in relationship to others (domain of social environment) and perspective of the future (domain of future perspective). Suffering in these domains, related to disease as well as independent of disease, adds to suffering and needs to be investigated [30, 31]. The meaning of suffering [21, 30–36] cannot be fully understood by a study limited to providing scores. Assessment about the personal experience of the suffering is necessary.

A framework of categories of suffering may help to organize qualitative study outcomes of experiences of suffering. The qualitative descriptions may be attributed to a framework of biomedical, psychodynamic and emotional categories of suffering. An important psychodynamic perspective of suffering is the concept of loss, such as loss of meaning, loss of autonomy, loss of dignity or loss of hope [21, 31, 33, 34, 36, 37]. Emotional categorizations of suffering include depression, hopelessness, demoralization, anxiety, worrying and feeling tensed [31, 38–40]. Suffering is complex and there is no universal, clear-cut, comprehensive system of categorization of suffering [31, 41]. The frequently employed categorization in physical, psychological, social and existential suffering [32, 42–45] demonstrates overlap. This categorization may suggest that existential suffering is a separate entity. However, physical, psychological and social suffering may result in existential suffering, and existential suffering may be part of various categories of suffering [46]. For the purpose of analysis of qualitative data about suffering a considered choice may be made for categories relevant to the study perspective.

### Measurement instrument: the State-of-Suffering V

To realize this study the State-of-Suffering V (SOS-V) was developed. The SOS-V is a structured, quantitative instrument for comprehensive assessment of unbearable suffering related to symptoms, with additional open ended questions to investigate the experience of suffering [17, 25]. “Symptoms” refers to physical, psychological, social and existential aspects of suffering; this extended interpretation of symptoms is not uncommon in psychosomatic research. Cancer is polysymptomatic [26] and systematic assessment of symptoms is indicated [27]. Based upon literature study a framework of domains in which suffering may occur was selected and symptoms relevant to end-of-life cancer populations were introduced. The SOS-V systematically addresses 69 symptoms in a framework of five domains: (I) medical symptoms; (II) loss of function; (III) personal aspects; (IV) environment and; (V) nature and prognosis of disease [25]. For every symptom two questions are asked. First, what is the intensity (or extent) of the symptom? Second, if the symptom is present, to what extent does the symptom cause unbearable suffering?

A uniform 5-point scoring scale with a description is employed for both questions: 1-not at all; 2-slightly; 3-moderately; 4-seriously; 5-very seriously, hardly could be worse. When a patient rates 4 or 5 for unbearable suffering, the experience is further explored through open ended questions. Answers are immediately written down as quoted phrases. After rating all 69 symptoms the interviewer asks whether individual aspects of suffering are missing, and if so documents and rates these as well. Reduction of symptoms investigated with the SOS-V was not striven for; suffering is multidimensional, the symptoms are clinically differentiable and it requires consideration that even one symptom may determine unbearability. The total number of symptoms which cause suffering does not automatically add up to an overall experience of suffering. The overall experience of suffering is assessed at the end of the interview; the patient is asked to consider all present symptoms and rate overall unbearable suffering (same scale). The two days before the interview were the reference period for assessment of suffering. The development of the instrument is described elsewhere [25]. Administration of the quantitative section of the SOS-V generally was possible within 15 to 20 minutes [12, 25].

### Analysis

Only the final SOS-V interviews were analyzed as they were the interviews closest to death, taking in account that performing EAS in response to an explicit request in majority occurs in the final two weeks of life [13, 16]. The question about the intensity of symptoms was dichotomized into the symptom not at all being present (rating 1) versus the symptom being present (ratings 2–5). When a symptom was not at all present, it was assumed that the symptom did not lead to unbearable suffering, and a rating of 1 was given for unbearable suffering. After that the quantitative data about unbearable suffering were analyzed dichotomously. Ratings 1 (not at all), 2 (slightly) or 3 (moderately) for suffering were defined as bearable; ratings 4 (seriously) or 5 (very seriously, hardly could be worse) were defined as unbearable. Differences in prevalence of unbearable symptoms and overall unbearable suffering between patients who did and who did not request EAS were tested with a Fisher’s exact test for nominal variables and with a t-test for continuous variables; the p-level was 0,05. Additionally Bonferroni analysis for high numbers of independent tests was applied.

To organize the qualitative descriptions of the experiences of suffering, related to symptoms for which the patients rated 4 or 5 for unbearable suffering, a categorization schedule was composed of senses of suffering which are relevant in patients at the end of life (Table 1) [17]. Existential suffering, an important construct of suffering, was not separately identified, from the perspective that other identifiable senses of suffering all may potentially contribute to existential suffering [21, 26–30, 32–36, 38, 41–43, 46–48]. Two raters, a GP (CR) and an external clinical psychologist, independently rated the descriptions of the experiences of unbearable suffering according to the categorization schedule. One rating per unbearable description was permitted. The raters then deliberated about the ratings which were not identically attributed and provided identical rating, if possible, after discussion. Only identically attributed ratings were used to analyze differences between patients who did and who did not request EAS. Consensus between the raters occurred in 86% of the analyzed qualitative descriptions. T-test for mean prevalence were used for statistical analysis. The rating process is described in more detail elsewhere [17].

**Table 1:** **Categorization of qualitative data**

Category of suffering	Indications for assigning category	Example of categorization
Physical	Medical morbidity, the physical symptom itself, physical symptoms which result in physical experienced suffering	*Pain*: I have pain all day, it occupies me continuously, there is little distraction
Loss of meaning	Loss of: identity, capacity of self-fulfillment, communication, social role, social interaction, intimacy	*Impaired working capacity*: I miss the contact with people. The only one left is my wife
Loss of autonomy	Suffering acknowledged to be caused by loss of autonomous functioning and occurrence of dependency (presence itself of loss of autonomy is not sufficient for assigning)	*Trouble accepting present situation*: The dependency on other people
Loss of dignity	Socially embarrassing symptoms, shame, body image concerns, not taken seriously, worthlessness	*Impaired comprehension of speech*: I feel stupid, I can’t come along
Burden to others	Experience to be a burden to others	*Feeling to be a burden to others*: I feel troubled to ask other people for help
Loss of sexual role	Loss of capability of sexual functioning; loss of sexual role	*Restricted sexuality*: my sexual life is destroyed, it is gone, not only for me, but also for my wife
Fear of future suffering	Fear caused by awareness of possible suffering related to progress of disease	*Fear to lose the strength to bear the suffering*: Pain, I have fear for pain
Anxiety	Anxiety	*Not sleeping well*: I have these nightmares, it wakens me up and makes me frightened
Death anxiety	Anxiety related to awareness of the process of dying and what may come along with that, and anxiety related to the actual dying process	*Fear of future suffering*: I am afraid to suffocate
Depressiveness	Suffering caused by the presence of depressive thoughts	*Feel depressed*: It is an annoying feeling. It is also influenced by the situation of my daughters: they are not doing well
Worrying	Negative thoughts which cannot be turned off	*Impaired coordination of movements*: I am afraid to fall, I hold on to everything
Feeling tensed	Feeling tensed in mind or body	*Feel tensed*: Especially in bed; each time again I feel tense and keep saying “relax”
Hopelessness	Loss of possibility of meaning	*Hopelessness*: To say farewell is what makes me feel hopeless
Pointlessness	Total loss of meaning; nothing left	*General discomfort*: Not being able to do a thing, just waiting for death

## Results

The studied sample consisted of the 64 patients who died during follow-up; 46 patients died within six months after inclusion. The average age was 70 years (range 38–86), 52% were female, all patients were Caucasian. Lung cancer (27%) and gastro-intestinal cancer (25%) were most prevalent. An explicit request for EAS occurred in 27% (17 patients); EAS was performed in 8% (5 patients). A part of the study design was to obtain information from GPs that the condition of a patient had rapidly deteriorated, so that additional interviews could be planned close to death. This rarely occurred. The final interview was on average 30 days before death (SD 17 days); in 23% the interview was administered within two weeks prior to death. An advance euthanasia directive (77% versus 9%) and higher education (defined as any further education after high school) (35% versus 13%) were significantly more frequent in the group of patients with an explicit request; for the other demographic characteristics no differences occurred (Table 2). The prevalence of explicit requests for EAS was 15% (16 out of 110 patients) in the sample which was not invited to participate, and 10% (7 out of 72 patients) in the sample which declined participation.

**Table 2:** **Patient characteristics for patients with and without an explicit request for euthanasia (n = 64)**

	Explicit request	No explicit request	p-value*
	n = 17 % (n)	n = 47 % (n)	
Age (mean(standard deviation)	71.9(10.2)	69.3(12.5)	0.437
Male	59(10)	45(21)	0.400
Partner status: single	53(9)	68(32)	0.265
Education			0.043
- Higher	35(6)	13(6)	
- Middle or lower	65(11)	87(41)	
Religious^†^	63(10)	62(29)	0.955
Advance euthanasia directive	77(13)	9(4)	<.001
Type of cancer			0.738
- Lung	35(6)	30(14)	
- Colorectal	24(4)	15(7)	
- Hematologic	6(1)	11(5)	
- Urologic	12(2)	9(4)	
- Breast	0(0)	9(4)	
- Other	24(4)	28(13)	

Rounded percentages and absolute numbers.

*Fisher’s exact test for all variables except age (t-test).

^†^one missing observation (in group ‘explicit request for euthanasia’).

Unbearable symptoms were present in 88% (n = 53) of the 60 patients studied with the SOS-V. No significant differences in prevalence of unbearable symptoms occurred between patients with and without an explicit request for EAS (Tables 3 and 4). “Needing help with housekeeping” was unbearable more frequently in patients with an explicit request for euthanasia (65% versus 33%), however after application of the Bonferroni correction no significant differences remained. No differences occurred between patients with and without an explicit EAS request for mean total of unbearable symptoms (mean 11.6 ± SD 8.6 unbearable symptoms versus mean 10.3 ± SD 9.3 unbearable symptoms) and for prevalence of overall unbearable suffering (33% versus 28%). Unbearable symptoms were present in 94% of patients with an explicit request for EAS and in 87% of patients without an explicit request. There were no differences in numbers of unbearable symptoms per domain.

**Table 3:** **Symptom unbearability in the domain of medical symptoms in patients with and without an explicit request for euthanasia (n = 60)**

	Symptom present %(n)*	Symptom unbearable			
		EAS request (n = 17) %(n)	No EAS request (n = 43) %(n)	P-value	All patients (n = 60) % (n)*
Domain I: Medical symptoms					
Weakness	93 (56)	71 (12)	51 (22)	0.249	57 (34)
General discomfort	80 (48)	35 (6)	37 (16)	0.995	37 (22)
Tiredness	87 (52)	47 (8)	30 (13)	0.243	35 (21)
Pain	72 (43)	29 (5)	23 (10)	0.743	25 (15)
Loss of appetite	62 (37)	18 (3)	28 (12)	0.520	25 (15)
Not sleeping well	47 (28)	29 (5)	23 (10)	0.743	25 (15)
Changed appearance	78 (47)	18 (3)	23 (10)	0.740	22 (13)
Vomiting	27 (16)	24 (4)	19 (8)	0.726	20 (12)
Shortness of breath	59 (35)	24 (4)	16 (7)	0.712	19 (11)
Impaired co-ordination	57 (34)	12 (2)	21 (9)	0.713	18 (11)
Loss of concentration	40 (24)	12 (2)	19 (8)	0.711	17 (10)
Memory loss	43 (26)	12 (2)	16 (7)	0.995	15 (9)
Incomprehensible speech	32 (19)	18 (3)	14 (6)	0.704	15 (9)
Nausea	28 (17)	12 (2)	14 (6)	0.725	13 (8)
Smelling unpleasant	35 (21)	18 (3)	12(5)	0.676	13 (8)
Impaired hearing	33 (20)	12 (2)	14 (6)	0.996	13 (8)
Thirst	45 (27)	18 (3)	9 (4)	0.399	12 (7)
Feeling tensed	44 (26)	24 (4)	7 (3)	0.090	12 (7)
Impaired mental clarity	42 (25)	12 (2)	12 (5)	0.995	12 (7)
Swallow food impaired	35 (21)	12 (2)	12 (5)	0.995	12 (7)
Feeling depressed	34 (20)	6 (1)	14 (6)	0.661	12 (7)
Constipation	30 (18)	18 (3)	9 (4)	0.393	12 (7)
Dizziness	27 (16)	0 (0)	16 (7)	0.175	12 (7)
Hiccups	22 (13)	18 (3)	7 (3)	0.338	10 (6)
Intestinal cramps	22 (13)	18 (3)	5 (2)	0.132	8 (5)
Impaired sight	42 (25)	12 (2)	5 (2)	0.317	7 (4)
Itch	32 (19)	12 (2)	5 (2)	0.317	7 (4)
Feeling anxious	27 (16)	6 (1)	7 (3)	0.997	7 (4)
Swallowing fluid impaired	23 (14)	12 (2)	5 (2)	0.317	7 (4)
Diarrhea	20 (12)	12 (2)	5 (2)	0.317	7 (4)
Incontinence of feces	8 (5)	6 (1)	7 (3)	0.997	7 (4)
Coughing	38 (23)	6 (1)	5 (2)	0.997	5 (3)
Pressure ulcers	8 (5)	12 (2)	0 (0)	0.996	3 (2)
Comprehension of speech impaired	7 (4)	0 (0)	5 (2)	0.998	3 (2)
Paralyzed limbs	5 (3)	0 (0)	2 (1)	0.999	2 (1)
Skin metastasis	3 (2)	0 (0)	2 (1)	0.999	2 (1)
Incontinence of urine	10 (6)	0 (0)	0 (0)	1.00	0 (0)

The SOS-V was missing in four patients.

Scoring: 1-not at all; 2-slightly ; 3-moderately; 4-seriously; 5-very seriously, hardly could be worse.

Suffering bearable: scores 1–3; Suffering unbearable: scores 4,5.

Rounded percentages and absolute numbers.

*Between 0 to 1 missing observations per symptom.

Fisher’s exact tests.

**Table 4:** Symptom unbearability in the domains of loss of function, personal aspects, environment, nature and prognosis of disease in patients with and without an explicit request for euthanasia (n = 60; for patients with administered SOS-V)*

	Symptom present	Symptom unbearable			
		EAS request	No EAS request	p-value**	All patients
	%(n)	(n = 17) %(n)	(n = 43) %(n)		(n = 60) %(n)
*Domain II: Loss of function*					
Impaired routine daily activities	83 (50)	53 (9)	56 (24)	0.995	55 (33)
Impaired leisure activities	82 (49)	65 (11)	44 (19)	0.252	50 (30)
Help needed with housekeeping	71 (42)	65 (11)	33 (14)	0.040**	42 (25)
Bedridden	56 (33)	29 (5)	33 (14)	0.996	32 (19)
Help needed with self-care	60 (36)	35 (6)	16 (7)	0,192	22 (13)
Impaired working capacity	17 (10)	6 (1)	14 (6)	0.661	12 (7)
Impaired sexuality	14 (8)	0 (0)	7 (3)	0.551	5 (3)
*Domain III: Personal aspects*					
Feeling dependent on others	80 (48)	47 (8)	44 (19)	0.995	45 (27)
Not able to do things you consider important	63 (36)	35 (6)	42 (18)	0.773	42 (24)
Trouble accepting the present situation	60 (36)	41 (7)	30 (13)	0.545	33 (20)
Loss of control over your own life	30 (18)	18 (3)	30 (13)	0.518	27 (16)
Negative thoughts or worrying	32 (19)	6 (1)	19 (8)	0.423	15 (9)
Feeling a nuisance to others	38 (23)	24 (4)	9 (4)	0.206	13 (8)
Hopelessness	28 (17)	24 (4)	9 (4)	0.206	13 (8)
Feeling not any longer being the same person	28 (17)	0 (0)	14 (6)	0.170	10 (6)
Feelings of worthlessness	22 (13)	12 (2)	9 (4)	0.995	10 (6)
Feeling lonely (intrapersonal)	20 (12)	6 (1)	16 (7)	0.420	10 (6)
Experienced little happiness with family/friends	22 (13)	12 (2)	7 (3)	0.616	8 (5)
Feeling of no longer being important to others	18 (11)	12 (2)	7 (3)	0.616	8 (5)
Feeling tired of life	17 (10)	12 (2)	7 (3)	0.996	9 (5)
Not satisfied with own self	12 (7)	6 (1)	7 (3)	0.995	7 (4)
Feelings of guilt	12 (7)	6 (1)	5 (2)	0.490	5 (3)
Lived a life with little purpose	8 (5)	6 (1)	2 (1)	0.997	3 (2)
Experienced little success in life	10 (6)	0 (0)	2 (1)	0.236	2 (1)
*Domain IV: Environment*					
Relatives consider your suffering too severe	33 (19)	24 (4)	12 (5)	0..995	16 (9)
Practical loneliness (isolation, no one present for you)	15 (9)	12 (2)	12 (5)	0.995	12 (7)
Insufficient availability of care	12 (7)	12 (2)	7 (3)	0.616	8 (5)
Unsatisfactory social contacts	8 (5)	6 (1)	2 (1)	0.491	3 (2)
Insufficient support (family, relatives)	5 (3)	0 (0)	2 (1)	0.490	2 (1)
Shame	2 (1)	0 (0)	2 (1)	0.490	2 (1)
*Domain V: Nature and prognosis of disease*					
Fear of future suffering	40 (24)	24 (4)	14 (6)	0.453	17 (10)
Fear of future failing strength to bear suffering	25 (15)	6 (1)	12 (5)	0.662	10 (6)

The SOS-V was missing in four patients.

Scoring: 1-not at all; 2-slightly; 3-moderately; 4-seriously; 5-very seriously, hardly could be worse.

Suffering bearable: scores 1–3 ; Suffering unbearable: scores 4,5.

Rounded percentages and absolute numbers.

*0 to 3 missing observations per symptom. Impaired working capacity applied for 10 persons (with work).

**Fisher’s exact tests.

The qualitative analysis demonstrated no significant differences in senses of unbearable suffering between patients with and without an explicit request for euthanasia (Table 5). Examples of attributions of qualitative data to categories are additionally provided in Table 1. Additional information about the trajectory of patients with an explicit request for EAS is provided in Table 6.

**Table 5:** **Distribution of the qualitative data related to unbearable symptoms over categories of suffering in patients with and without an explicit request for euthanasia (n = 60)**

	Explicit request (n = 17)		No explicit request (n = 43)		
Category of suffering	Patients in whom the category of suffering was present % (n)	Number in which the category of suffering occurred per patient* Mean (SD)	Patients in whom the category of suffering was present % (n)	Number in which the category of suffering occurred per patient* Mean (SD)	p-value of t-test for means
Physical suffering	76 (13)	3.0 (2.3)	72 (31)	2.8 (2.7)	0.752
Loss of meaning	88 (15)	2.6 (2.0)	65 (28)	2.4 (2.8)	0.843
Loss of autonomy	76 (13)	1.9 (1.9)	49 (21)	1.6 (1.1)	0.571
Loss of dignity	35 (6)	0.8 (1.9)	42 (18)	1.6 (2.0)	0.847
Experience to be a burden to others	41 (7)	0.5 (0.7)	21 (9)	0.3 (0.5)	0.168
Loss of sexual role	0 (0)	0.0 (0.0)	5 (2)	0.05 (0.2)	0.374
Fear of future suffering	18 (3)	0.2 (0.4)	14 (6)	0.3 (0.7)	0.644
Anxiety	6 (1)	0.1 (0.2)	19 (8)	0.3 (0.8)	0.078
Death anxiety	6 (1)	0.1 (0.5)	0 (0)	0.0 (0.0	0.332
Depressive thoughts	6 (1)	0.1 (0.2)	7 (3)	0.1 (0.3)	0.881
Worrying	6 (1)	0.1 (0.2)	19 (8)	0.2 (0.5)	0.087
Feeling tensed	6 (1)	0.1 (0.2)	7 (3)	0.1 (0.3)	0.881
Hopelessness	6 (1)	0.1 (0.2)	7 (3)	0.1 (0.3)	0.881
Pointlessness	6 (1)	0.2 (1.0)	5 (2)	0.1 (0.5)	0.548

The SOS-V was missing in four patients.

*Only equal ratings were used for analysis.

**Table 6:** **Patient characteristics and end-of-life trajectories in patients with an explicit euthanasia request (N=17)**

Gender, age, tumor, euthanasia directive	End of life	Additional information provided by GP
Female, 76, colon, (+)	Euthanasia	Euthanasia in hospice by the GP; the patient did not want to continue living after witnessing death of 26 others.
Female, 76, eye tumor, (+)	Euthanasia	Died in hospice; the GP performed euthanasia.
Male, 76, M. Kahler, (+)	Euthanasia	The GP performed euthanasia at home when patient became exhausted after development of pneumonia. The GP stated: “the wish of the patient was provided”.
Female, 55, colon, (+)	Euthanasia	Euthanasia performed in hospice in other town.
Male, 85, colon , (+)	Physician assisted suicide	Died at home, ingestion of barbiturates.
Female, 71, lung, (+)	Terminal sedation	The euthanasia procedure had been initiated, all compulsory criteria were confirmed present, the final step towards EAS as yet was not set. The patient was terminal, became drowsy, and changed her mind, expressing to a visiting physician in out of regular hours care (during holidays) the wish to continue living, and not to perform EAS. One day later, after deliberation, terminal sedation was initiated. Then the family demanded euthanasia. The GP of the patient considered to perform euthanasia. The consulting physician opposed euthanasia, considering absent noticeable suffering. The patient died without signs of suffering. The family remained dissatisfied.
Male, 69, mesothelioma	Terminal sedation	Died at home.
Male, 44, renal, (+)	Terminal sedation	Died at home.
Male, 84, lung, (+)	Natural death	No mention of persisting request; died at home
Female, 80, Grawitz, (+)	Natural death	A former nun who requested euthanasia when suffering increased. The euthanasia procedure was initiated and all compulsory criteria were assessed to be present. Ultimately the patient decided on religious grounds not to continue the path of euthanasia and died at home.
Female, 79, esophagus, (+)	Natural death	No mention of persisting request; died at home.
Male, 78, lung, (+)	Natural death	No mention of persisting request; died in hospice cared for by the GP.
Female,78, colon	Natural death	No mention of persisting request; died in hospice cared for by the GP.
Male, 74, adenocarcinoma	Natural death	No mention of persisting request; died at home.
Male, 72, lung, (+)	Natural death	No mention of persisting request; died at home.
Female, 68, lung, (+)	Natural death	No mention of persisting request; died in hospice cared for by the GP.
Male, 66, lung	Natural death	No mention of persisting request; died at home.

(+): euthanasia directive present.

Two of the patients withdrew their request for EAS. Reasons why EAS was not performed could not be retrieved from the medical files in the other patients. No conflicts between patients and GPs about whether or not to perform EAS were identified; in one case a conflict with the family occurred because EAS was not performed. Referral to other physicians, because GPs did not agree with performing EAS, did not occur.

## Discussion

The present study touches on fundamental questions about suffering, autonomy and the tasks of the medical profession concerning life and death. We have not identified prior patient directed studies which prospectively investigated unbearable suffering in relationship to requests for EAS in a cohort of patients. EAS in one out of three patients with an explicit request for euthanasia is comparable to findings in other studies in the Dutch setting [13, 14, 49, 50].

No differences occurred in prevalence of unbearable symptoms, or in prevalence of overall unbearable suffering, between patients with and without an explicit request for EAS. Frequently mentioned motives for requesting EAS are loss of control, loss of autonomy, loss of dignity, not wanting to be a burden to others and fear of the future [8, 32, 51–55]. The prevalences of these motives, from the perspective of unbearable suffering, were not different for patients with and without a request for EAS. Loss of control, isolation, hopelessness, burden on others and fear about the future, which in research were found indicative of existential issues [46], were not different for patients with and without unbearable suffering. These findings may indicate that unbearable suffering is not the dominant motive to request EAS.

Unbearable symptoms occurred in 88% of this primary care population of end-of-life cancer patients in the period around one month before death. This indicates that the criterion of unbearable suffering, also when not being the decisive motive to request EAS, may be met in the majority of end-of-life cancer patients cared for in primary care when initiating the procedure of compulsory criteria assessment to evaluate whether EAS is permitted. How to interpret this finding as part of the process in which physicians respond to requests for EAS is unclear. Identified causes for not granting requests for EAS included death of the patient before performance of EAS (20%; percentage of total of ungranted requests), death of the patient before finalization of decision making (20%), withdrawal of the request by the patient (20%) and refusal of the physician to comply to the request of the patient (18%) [49].

Motives to request EAS may need to be interpreted from a different perspective. A desire to control the circumstances of one’s death, rather than unbearable suffering due to loss of control, may determine the request for EAS. Another driver of requests for EAS may be loss of the will to continue living [56–58], caused by loss of positive feelings towards life and (or) loss of connectedness with life [32, 36, 38, 47, 53, 57]. Loss of energy and exhaustion, related to tiredness and weakness which are prevalent among end-of-life cancer patients [29], may contribute to loss of the desire to continue living. Depression and related suicidality in cancer patients are possible causes of a request for EAS [59–61]. The prevalent role of depression related to requesting for EAS has not been assessed in research [62, 63]. Another perspective is that a genuine desire to die is absent in patients who express a wish to hasten death [22, 64]. The underlying message of a request for EAS might be a cry for help [22], a request to be given a reason to live [37, 64], or an expression of feelings [65]. None of these has been sufficiently recognized or addressed. Finally societal influences need to be considered. Individual choice is an important societal driver of Western society [66]. Practices which enhance autonomous choice in health care, e.g. physician initiated discussions of the subject of EAS at an early stage [67], or granting the wish of the patients as the motive to provide EAS [13], may influence direction and outcomes of provision of care [3, 19, 66, 68, 69]. The presence of an advance pro-euthanasia directive and higher education, which occurred more frequently in patients with an explicit request, may be in line with the perspective of control and with the perspective of choice.

Patients depend upon responses of physicians. In responding to patients with a request for EAS it needs to be realized that dying cancer patients perceive that they cannot feel completely independent, which affects true autonomous decision making [70, 71]. Additionally, processes of transference may influence the communication between patient and physician. Awareness of such processes is important when responding to patients with a request for EAS [70, 72, 73]. Physician factors in response to the suffering of patients include attitude and quality of provided palliative care [18, 23, 72, 74]. Palliative interventions to reduce suffering in end-of-life cancer patients include symptom reduction [28], psycho-oncologic interventions [32, 33, 36, 47, 75, 76], spiritual care [77, 78] and palliative sedation [79, 80]. A tradition of research investigating effectiveness of palliative interventions in primary care populations does not exist. Therefore little is known about the effectiveness of interventions to reduce the suffering of end-of-life cancer patients in this setting. Even less is known about the question of which interventions may reduce the prevalence of requests for EAS, or performance of EAS. A remarkable quote in a Dutch qualitative study was that some GPs, since adopting a more caring attitude, found they no longer had to perform euthanasia [74].

The present study has some limitations. The first is the limited number of patients, which limits statistical power. We cannot rule out the possibility of differences in unbearable suffering between patients with and without an explicit request that we did not find. Interviews with most patients were not in the final days of life; unbearable suffering may have progressed, or may have been adequately treated. Furthermore, for the patients who explicitly requested euthanasia, the interview was not administered at the time of the request. The sample concerns a Western population in primary care in a context of legally permitted EAS, which limits generalizability.

## Conclusions

We conclude that in a population of end-of-life cancer patients cared for in primary care no differences in unbearable suffering were found between patients with and without explicit requests for EAS. The study raises the question of whether unbearable suffering is the dominant motive to request for EAS. Another outcome was that most patients suffered from unbearable symptoms, indicating that the compulsory criterion of unbearable suffering, part of the criteria which permit performing EAS, may be met a priori in most end-of-life cancer patients dying at home, whether they request EAS or not.

### Ethical approval

The study protocol was approved by the Medical Ethics Committee at the VU University Medical Center (METC VUmc No. 2002/79).
